# Submaximal Testing to Estimate Aerobic Capacity Using a Matrix C5x Stepmill

**DOI:** 10.2478/hukin-2022-0055

**Published:** 2022-09-08

**Authors:** Lauren von Schaumburg, Kelly R. Laurson, David Q. Thomas, Kristen M. Lagally

**Affiliations:** 1 Illinois State University, 100 N University St, Normal, IL 61761, United States

**Keywords:** VO_2max_, METs, Watts, stair climbing, stepmill

## Abstract

The primary purpose of this study was to determine if the Matrix C5x stepmill’s preprogrammed submaximal test is able to accurately predict maximal oxygen uptake. Sixteen participants completed a maximal treadmill test and a preprogrammed submaximal test on a Matrix C5x stepmill. Oxygen uptake was measured using a Cosmed K5 during both tests. Maximal oxygen uptake (VO_2max_) was calculated from submaximal data using a multi-stage calculation and compared against measured VO_2max_ from the maximal test and estimated VO_2max_ from the submaximal stepmill test. METs were also measured during the submaximal test and compared to the METs estimated by the stepmill and METs calculated using submaximal stepping equations. Measured VO_2max_ (39.18 α 6.6 ml^.^kg^-1.^min^-1^) was significantly higher (p < 0.001) than estimated VO_2max_ (28.06 α 3.2 ml^.^kg^-1.^min^-1^) and calculated VO_2max_ (35.58 α 8.0 ml^.^kg^-1.^min^-1^). Measured METs were significantly (p = 0.04) higher than estimated METs in all stages, and higher than calculated METs in stage 1 of the submaximal test. The C5x did not provide accurate estimations of METs or maximal oxygen uptake. Calculating maximal oxygen uptake from submaximal stepmill data may provide an alternative, although development of a new equation may be warranted.

## Introduction

Assessment of maximal aerobic capacity (VO_2max_) has been studied extensively using treadmills and cycle ergometers (Noonan and Dean, 2000). Research has shown that the specificity of testing (i.e., testing on the mode used for training) has positive effects on the testing outcome (Stromme et al., 1977). Stair climbing using a climb mill or stair stepper has gained popularity as a training mode so it may be a useful mode with which to perform exercise testing, particularly for firefighters, hikers, and others who engage in sports or activities that include stair climbing motions.

There are conflicting results when VO_2max_ derived from a maximal StairMaster or a stepmill test is compared to VO_2max_ values obtained from other modes. Some studies found peak VO_2_/VO_2max_ to be higher when measured on a treadmill compared to a stair stepping device (Ben-Ezra and Verstraete, 1988; Luketic et al., 1993; Schuler et al., 1998). Other studies found no significant differences in peak VO_2_/VO_2max_ values between stair stepping devices and treadmills (Holland et al., 1990, 1988).

Submaximal testing may be more applicable than maximal testing in commercial fitness settings, however. Research examining the validity of submaximal stair climbing exercise on a StairMaster 4000PT to predict maximal oxygen uptake found conflicting results for trained and untrained women. Participants who had previously trained on a step ergometer and had previous experience with cardiovascular training had no significant differences in VO_2max_ values measured from a maximal Bruce treadmill test and those estimated from the StairMaster submaximal test. The non-step trained group had significantly lower VO_2max_ values when estimated from the StairMaster test than when measured during the maximal Bruce test (Roy et al., 2004).

Using a maximal StairMaster (model not specified) test, Carroll et al. (1990) developed an equation (VO_2max_ = 66.852 - .178(HR3) – 3.413(sex) [HR3: HR at 60 Steps/Minute, sex: men = 1, women = 2] to predict VO_2max_ from submaximal stepping exercise. When cross-validated with 27 men and women, the equation’s predicted VO_2max_ values were found to be strongly correlated (r = 0.71) with measured VO_2max_ assessed from the maximal StairMaster test. The results from these studies suggest that submaximal StairMaster exercise may have potential as a method for estimating peak/maximal oxygen uptake.

One concern about using submaximal stair climbing to predict maximal oxygen uptake is that a number of studies have found elevated physiological responses at standard submaximal levels in stair climbing when compared to other modes of exercise. For instance, two studies found lactate levels to be higher during exercise on stair climbing devices when compared to exercise on a treadmill (Schuler et al., 1998; Zeni et al., 1996). Higher VO_2_ and/or higher heart rates at similar workloads between a stair climbing device and a treadmill were found as well in other studies (Holland et al., 1990; Howley et al., 1992). Since submaximal testing typically relies on the heart rate to predict maximal oxygen uptake, an elevated heart rate at a given submaximal level will lead to a lower VO_2max_ prediction. Additionally, higher lactate levels and VO_2_ may lead to greater discomfort during stair climbing tests when compared to treadmill tests. These responses may limit the ability to accurately predict VO_2max_ from a submaximal step climbing test.

Additionally, there is evidence that step climbing machines may not estimate MET levels accurately. Some studies found METs to be overestimated by a step climbing device when compared to measured METs (Holland et al., 1988; Howley et al., 1992; Luketic et al., 1993). Conversely, Butts et al. (1993) found that at all stepping rates but the slowest, MET values were underestimated by a StairMaster 4000PT. If the stair climbing device uses intensity to calculate predicted VO_2max_ but cannot predict METs accurately, it cannot predict VO_2max_ accurately.

While the StairMaster 4000 PT commonly examined in previous research is not in production any more, StairMaster and other commercial gym equipment manufacturers are producing stepmills or climb mills (StairMaster, 2019). One of these companies, Matrix, provides a pre-programmed submaximal testing protocol on their C5x climb mills (Matrix Fitness, 2012). The purpose of this study was to examine how the submaximal prediction of VO_2max_ from the Matrix C5x compares to VO_2max_ derived from a maximal test on a treadmill. A secondary purpose was to compare measured MET values to those estimated by the Matrix C5x.

## Methods

### Participants

Eighteen participants were recruited on a campus from various classes or by word of mouth. Only sixteen participants were included in the analyses, however, because one was eliminated due to not meeting the criteria for reaching maximal effort and another was lost due to error in data collection. Each participant completed a medical history form and informed consent before participating in the study. Participants were excluded if they were not free from existing cardiovascular, metabolic and renal disease and symptoms suggestive of those diseases. They were also excluded if they had any musculoskeletal injury, or were not at least 18 years of age. This study was approved by the Institutional Review Board of the University.

### Design and Procedures

Each participant took part in two tests, a submaximal stepmill test and a maximal treadmill test. The two tests were performed on different days in random order, which was determined by rolling a die. Both tests were explained prior to testing, as was the Borg 6-20 ratings of perceived exertion (RPE) scale. Resting blood pressure was taken before each test and the RPE, heart rate, and exercise blood pressure were taken throughout the maximal test. The heart rate and RPE were measured throughout the submaximal test. The heart rate was measured via telemetry using a Polar E600 heart rate monitor and oxygen uptake was measured throughout both tests using a K5 portable metabolic system, which was calibrated according to manufacturer’s instruction prior to each test (COSMED, Rome, Italy). Respiratory data were averaged for both of the tests into 30 s intervals.

### Measures – Maximal Treadmill Test (measured VO_2max_)

The maximal treadmill test utilized the Bruce protocol. The results from the maximal treadmill test served as the criterion measured VO_2max_ (MeasVO_2max_) value. Participants were instructed to continue to the point of volitional fatigue. The treadmill test was determined to be maximal if participants achieved at least two of the following criteria: a heart rate within α 10 beats/min of the age-predicted maximal heart rate, an RER > 1.1, and RPE > 17, or a plateau in VO_2_ (change of less than 150 ml.min^-1^ with an increase in exercise intensity). As mentioned above, one participant did not meet at least two of these criteria, and thus was removed from the data analysis.

### Measures – Submaximal Stepmill Test (estimated VO_2max_)

The stepmill test was a pre-programmed submaximal protocol on the Matrix C5x climb mill (Matrix Fitness, Wisconsin, United States), which has revolving steps that are 8 inches (.2032 m) in height. As indicated in the Matrix manual and on the machine’s display, the submaximal test is “a four stage test lasting three to five minutes, where the speed is increased until the HR is held between 115-150 bpm for two of the stages” (Matrix Fitness, 2012). As such, the length of the test was assumed to be dependent on heart rate response, with the test ending when the heart rate was between 115-150 bpm for two stages in order for a prediction to be made by the stepmill. The C5x determined the heart rate by interfacing with the heart rate monitor. Investigators also used a separate Polar E600 watch to confirm accuracy of heart rates measured through the C5x.

Pilot testing of the Matrix submaximal test revealed several issues, including that on occasion and seemingly randomly, 1) stage length was inconsistent, 2) the steps per minute reported by the C5x were inaccurate, and 3) the C5x provided some participants with only one test stage and indicated that the test had “failed” due to “lack of the increase in the heart rate”. Specifically, after participants completed one stage, the Matrix C5x displayed a message stating “No Increase in the Heart Rate. Retry the test”. Nevertheless, when this message appeared, the C5x display still provided a predicted value for VO_2max_. All “successful” C5x tests consisted of two stages after which a predicted VO_2max_ value was provided on the C5x display. The values provided by the C5x stepmill, whether a test failed or succeeded, are reported here as estimated VO_2max_ (EstVO_2max_).

The Matrix company was contacted regarding the inconsistent testing procedures, and the Global Assistant Product Manager at Matrix provided the following equation [VO_2max_ = ((watts*12) + (weight(kg)*3.5))/weight(kg)] as the calculation used by the Matrix C5x to predict VO_2max_. Investigators intended to manually calculate EstVO_2max_ for both failed and successful tests. Unfortunately, it was unclear what value from the test should be used for Watts, and further attempts at communication with Matrix about this issue did not yield any information. Investigators attempted to complete the calculation using both the final Watts and average Watts reported by the C5x as part of the submaximal stepmill test. However, neither value yielded reasonable results for either METS or VO_2max_ in ml.kg^-1.^min^-1^. Calculating VO_2max_ using the Matrix equation was further complicated by the fact that in “successful” tests, the Matrix C5x reported the same Wattage for both of the completed stages, despite differing step rates.

### Measures – Maximal oxygen uptake calculations (calculated VO_2max_)

The original intent of the study was to compare two VO_2max_ values, those estimated by the Matrix C5x (EstVO_2max_) and those measured by indirect calorimetry (MeasVO_2max_). Given the uncertainty surrounding the VO_2max_ calculation and testing procedures provided by Matrix, VO_2max_ was also calculated using the multi-stage model equation (Gibson et al., 2018; Roy et al., 2004) to provide an alternative to the Matrix C5x VO_2max_ estimation. This equation calculates the slope as the ratio of the differences between the two submaximal VO_2_ values and the associated HR values from the final two stages of the test, and estimates VO_2max_ from the following equation: VO_2max_ (ml.kg^-1.^min^-1^) = VO_2_ from final stage + slope (HR_max_ – HR from final stage) (Gibson et al., 2018; Roy et al., 2004).

To ensure accurate data for this calculation, investigators: 1) ensured achievement of a steady state heart rate (α 5 bpm in the last two minutes of a stage), 2) manually counted steps per minute during each stage, and 3) if a test failed, manually continued the test by increasing intensity until the participant achieved the target heart rate range of 115 - 150 bpm for two consecutive stages, as suggested by the Matrix manual. These procedures and target heart rate range are also generally accepted as appropriate for submaximal testing (American College of Sports Medicine et al., 2018). The maximal heart rate was calculated as the age-predicted maximal heart rate using the equation 208 - .7(age) (Tanaka at al., 2001).

In order to complete the multi-stage equation, submaximal VO_2_ from the two final test stages must first be determined either through estimation or measurement. Typically, ACSM Metabolic equations (American College of Sports Medicine et al., 2018) would be used to calculate estimated submaximal VO_2_ values (Gibson et al., 2018; Roy et al., 2004). However, the ACSM stepping equation assumes both an “up” and “down” component to stepping, which may not be appropriate for use with a revolving stepmill (Holland et al., 1988). As such, a manually counted step rate from the final two stages of the stepmill submaximal test (regardless of whether the machine indicated “passed” or “failed”) was used to calculate submaximal VO_2_ using the following equation provided by Holland et al. (1990), whose work also examined a revolving stepmill: [VO_2_ (ml.kg^-1.^min^-1^) = (Steps/min)(Step Height in m) X 2 + 3.5]. The calculated estimated submaximal VO_2_ values from the Holland et al. (1990) equation were then used in the multi-stage equation to calculate VO_2max_. The VO_2max_ results from these calculations are reported as the calculated VO_2max_ (CalcVO_2max_) value.

### Measures – METs

As mentioned above, Cosmed K5 metabolic equipment was used to measure submaximal VO_2_ during the stepmill test. VO_2_ in ml.kg^-1.^min^-1^ was divided by 3.5 to determine measured METs (METs_meas_) from submaximal stages performed in the stepmill test. METs reported by the Matrix C5x (METs_est_) and (METs_calc_), which are VO_2_ results calculated from the Holland et al.’s (1990) equation divided by 3.5, were also examined.

### Statistical Analysis

To examine if differences existed between participants for whom the stepmill test failed and those for whom it succeeded, a two-way mixed ANOVA was conducted to examine the effect of group (pass vs. fail on the stepmill test) on EstVO_2max_ and MeasVO_2max_. Significant interactions and main effects were probed using follow-up *t*-tests. For all sixteen participants, MeasVO_2max_ values were compared with EstVO_2max_ values and CalcVO_2max_ values with a one-way repeated measures ANOVA. A significant main effect was probed using follow-up paired samples *t*-tests. For all sixteen participants, METs_meas_ were compared to METs_est_ and METs_calc_ with separate one-way ANOVAs performed for each stage of the submaximal stepmill test. A significant main effect was probed using follow-up paired samples *t*-tests. Pearson correlations were calculated between the VO_2max_ and MET variables. The alpha level was set at *p* < 0.05. SPSS software was used to perform all analyses.

## Results

The mean height of participants was 171.2 cm (α 7.2). The mean mass was 69.1 kg (α 9.7), and the mean resting heart rate was 81 beats per minute (bpm) (α 14.2). Participants’ descriptive data are shown in Table 1. Means and standard deviations for maximal oxygen uptake are provided in Table 2.

**Table 1:** Descriptive statistics (N=16)

Variable	Mean ± SD	Range
Height (cm)	171.2 ± 7.2	160.0–184.2
Mass (kg)	69.1 ± 9.7	53.4–87.3
Age (years)	19.6 α 1.6	18–23
RHR (bpm)	81 ± 14.2	64–106
APMHR (bpm)	194.3 ± 1	192–195
Measured Maximal HR (bpm)	191.4 ± 9.3	178–210
VO_2max_ (ml.kg^-1^.min^-1^)	39.2 ± 6.6	30.5–51.3

RHR = resting heart rate APMHR = age-predicted maximal heart rate (208-.7(age)) VO_2max_ = VO_2max_ measured during the Bruce treadmill test

**Table 2:** VO_2max_ estimated by the C5x stepmill, measured from the maximal treadmill test, and calculated using the multi-stage model equation (N=16).

Group/mode	Mean VO_2max_ (ml.kg^-1^.min^-1^)	Range
EstVO_2max_	28.1 ± 3.2^b^	24.0–33.0
MeasVO_2max_	39.2 ± 6.6^a^	30.5–51.3
CalcVO_2max_	35.6 ± 8.0^a, b^	26.1–58.9

a = significantly different from C5x at p < .05 b = significantly different from measured VO_2max_ CalcVO_2max_= Submaximal VO_2_ from the final two stages of the submaximal step test were calculated using the equation provided by Holland et al. (1990). VO_2max_ was then calculated using the multistage model equation (Gibson et al., 2018; Roy et al., 2004).

For 44% of the participants (n = 7), the Matrix C5x stepmill reported a successful prediction of VO_2max_. The mean EstVO_2max_ for these seven participants was 28.6 α 3.5 ml.kg^-1.^min-^1^, while the mean MeasVO_2max_ was 35.1 α 4.1 ml.kg-^1.^min^-1^. For the other nine participants, the Matrix C5x stepmill reported that the submaximal test had failed. Using the value provided by the C5x, the mean EstVO_2max_ for the nine participants for whom the test failed was 27.7 α 3.2 ml.kg^-1^.min^- 1^and the mean MeasVO_2max_ was 42.3 α 6.7 ml.kg^-1.^min^-1^. These nine participants were provided with additional stages of submaximal exercise following the one-stage C5x test failure, in order to collect data for CalcVO_2max_. Four participants required three stages and one required four stages to achieve heart rates within the target range of 115-150 bpm. The remaining participants achieved target heart rates in two stages.

A two-way mixed ANOVA revealed no significant interaction (*p* = 0.54) between the method of determining VO_2max_ (Est vs. Meas) and group (Pass vs. Fail on stepmill test). There was a significant main effect of VO_2max_ method (*p* < 0.01, partial eta squared = 0.69). There was also a significant main effect of group (*p* = 0.04, partial eta squared = 0.28). Follow-up *t*-tests found significant differences in MeasVO_2max_ and EstVO_2max_ for both the group that had a failed test (*p* = 0.001) and the group that had a successful test (*p* = 0.047). There was also a significant difference (*p* = 0.03) between the passed and failed groups with regard to MeasVO_2max_ (35.1 vs. 42.3 ml.kg^-1.^min^-1^), but no significant difference in EstVO_2max_ (28.6 vs. 27.7 ml.kg^-1.^min^-1^).

Given no difference in EstVO_2max_ between participants whose stepmill tests had passed or failed, all 16 participants’ data were pooled for further analyses. A repeated-measures ANOVA revealed a significant main effect for the method of determining VO_2max_ at the *p* < 0.05 level (*p* < 0.001), with an overall large effect size calculated using eta squared of 0.54. Follow up paired *t*-tests revealed that MeasVO_2max_ (39.2 α 6.6 ml.kg^-1.^min^-1^) was significantly higher (*p* < 0.01, Cohen’s d = 1.69) than EstVO_2max_ (28.06 α 3.2 ml.kg^-1.^min^-1^). The mean CalcVO_2max_ (35.6 α 8.0 ml.kg^-1^.min^-1^) was significantly lower (*p* = 0.027, Cohen’s d = 0.55) than the mean MeasVO_2max_ and significantly higher (*p* = 0.003, Cohen’s d = 0.94) than the mean EstVO_2max_.

Means and standard deviations for MET values are shown in Table 3. Submaximal MET values were obtained using three methods: reported by the C5x stepmill (METS_est_), measured using indirect calorimetry (METS_meas_), and calculated from step height and a stepping rate using the Holland et al.’s (1990) equation (METS_calc_). METs derived in these three ways were compared for the first three stages of the submaximal step mill test using separate one-way ANOVAs. A significant main effect was found for each stage, and follow-up paired *t*-tests revealed a significant difference between METs_est_ and METs_meas_ in all three stages – Stage 1 (*p* < 0.01, Cohen’s d = 1.7), Stage 2 (*p* = 0.03, Cohen’s d = 0.78), and Stage 3 (*p* = 0.04, Cohen’s d = 1.42). In all three stages, MET values were significantly underestimated by the C5x. There was also a significant difference between METs_est_ and METs_calc_ in all three stages – Stage 1 (*p* < 0.01, Cohen’s d = 0.83), Stage 2 (*p* < 0.01, Cohen’s d = 1.79), and Stage 3 (*p* < 0.01, Cohen’s d = 2.02) - with METs_est_ being lower in each stage. There was no significant difference between METs_meas_ and METs_calc_ in stages 2 and 3, but METs_calc_ were significantly (*p* = 0.04, Cohen’s d = 0.58) lower in stage 1.

**Table 3:** METs reported by the C5x, measured using indirect calorimetry, and calculated (Holland et al., 1990) during the first three stages of the submaximal stepmill test.

Stage of submaximal test	METS_est_	METS_meas_	METS_calc_	N
Stage 1	4.0 ± .0^a, b^	5.3 ± .7	4.8 ± .0^a^	16
Stage 2	5.2 ± .5^a, b^	6.1 ± 1.1	6.0 ± .4	16
Stage 3	6.1 ± .5^a,b^	7.7 ± 1.1	7.2 ± .5	5

a = significantly different from measured METs at p < .05 b = significantly different from calculated METs at p < .05

The correlations between MeasVO_2max_ and the two VO_2max_ prediction methods revealed a significant correlation between MeasVO_2max_ and CalcVO_2max_ (r = 0.70, *p* = 0.003) (Gibson et al., 2018; Roy et al., 2004). The correlations between MeasVO_2max_ and EstVO_2max_ (r = -0.40, *p* = 0.121) and EstVO_2max_ and CalcVO_2max_ (r = 0.07, *p* = 0.808) were not significant.

Correlations among METs_meas_, METs_est_, and METs_calc_ were also determined. In Stage 1, the C5x step mill reported all participants’ METs_est_ as 4.0. As such, a correlation could not be determined for this stage. In stages 2 and 3, METs_est_ and METs_calc_ were strongly and positively correlated. In stage 2, the correlation was r = 0.96 (*p* < 0.001) and in stage 3 the correlation was r = 0.98, (*p* = 0.004). METs_meas_ and METs_calc_ were moderately correlated (r = 0.53, *p* = 0.04) in stage 2. However, in stage 3, the correlation between METs_meas_ and METs_calc_ was not significant (r = 0.11, *p* = 0.86). The correlations for METs_est_ and METs_meas_ were not significant in stages 2 or 3 (*p* = 0.09 and *p* = 0.82, respectively). Correlations for stage 4 were not calculated as only one participant completed four stages of exercise during the submaximal stepmill test.

## Discussion

The main purpose of this study was to compare directly measured VO_2max_ values to those predicted by a preprogrammed submaximal test performed on the Matrix C5x stepmill. Our results indicate that the C5x significantly under predicted VO_2max_ values. The mean value reported by the Matrix C5x was 28.1 ml.kg^-1.^min^-1^, which is considered “very poor to poor” for participants in this age range (American College of Sports Medicine et al., 2018), compared to the measured VO_2max_ value of 39.2 ml.kg^-1.^min^-1^, which is “poor to fair”. The C5x underestimated VO_2max_ for 13 of the 16 participants, with a mean difference of 13.1 α 6.8 ml.kg^-1.^min^-1^. Therefore, an individual evaluating their fitness using a Matrix C5x is likely to receive a discouraging and misleading result. Moreover, for over half of the participants, the submaximal test on the C5x “failed”, and so many users could feel compelled to perform the test again. Interestingly, VO_2max_ provided in spite of a “failed” test was not significantly different than that for a “successful” test, and VO_2max_ values were significantly underpredicted by the C5x across the board. One interesting finding was that participants for whom the test failed had a higher measured mean VO_2max_ value than those that had successful submaximal tests on the C5x. Of these nine participants, five needed more than two exercise stages (investigators manually provided additional stages when a test failed) to achieve target heart rates between 115-150 bpm. This, along with higher VO_2max_ values, is suggestive of higher cardiovascular fitness. The “failed test” error message from the C5x indicated “no increase in the heart rate”, which might suggest that the participant had not achieved the minimum baseline heart rate of 115 bpm and that this particular submaximal test is not suitable for more fit participants. However, four of the nine “failed” participants had heart rates above 115 bpm in the first stage of the C5x submaximal test. These inconsistencies make it difficult to draw conclusions about the Matrix C5x preprogrammed submaximal test.

With the exception of the equipment used (StairMaster vs. stepmill), our findings are almost identical to those of Roy et al. (2004) who found that a submaximal test performed using a StairMaster 4000PT resulted in a significant underestimation of VO_2max_ in non-step trained participants. The average measured VO_2max_ value for the non-step trained participants in their study was 37.6 ml.kg^-1.^min^-1^. StairMaster predicted VO_2max_ was 30.9 ml.kg^-1.^min^-1^, which was significantly lower than VO_2max_ measured during a maximal Bruce treadmill protocol. However, VO_2max_ values from the submaximal StairMaster test and the maximal treadmill test did not differ for step-trained participants (Roy et al., 2004).

Most of the previous research has been performed using a StairMaster (primarily the 4000 PT) rather than a stepmill device with revolving stairs, as was used in the present study. The research that has been done using a stepmill similar to the C5x has only used it for maximal exercise testing. For instance, Holland et al. (1990) found no significant differences in peak oxygen uptake (VO_2_), heart rate (HR), ventilation (Ve) or respiratory quotient (RQ) obtained from a maximal test on a treadmill compared to a maximal test on a “steptreadmill” (StairMaster 6000). Ben-Ezra and Verstraete (1988) completed a study with firefighters comparing a maximal treadmill test to a maximal test on a StairMaster 5000 (revolving stairs of eight inches) and found that VO_2max_, HR_max_, and VE_max_ were all lower from the StairMaster 5000 test. No previous studies have examined submaximal estimation of VO_2max_ using a step mill.

Another purpose of this study was to compare measured METs to those reported by the C5x during each submaximal workload. Results indicated that the C5x underestimated METs in all stages. This finding is similar to that of Butts et al. (1993) who found that the StairMaster 4000PT significantly underestimated METs. A majority of the previous studies found the opposite result, however. Using a stepmill device, Holland et al. (1988) found significant differences between measured MET values and device-reported MET values in a study with coronary heart disease patients. During the last two stages of a maximal test (measured intensity = 5.8 and 7.2 METs), they found an overestimation of METs by a StairMaster stepmill, although in the first two stages (measured intensity = 3.4 and 4.7 METs), the values between stepmill-reported and measured METs were similar (Holland et al., 1988). Howley et al. (1992) found MET values reported by a StairMaster 4000PT to be 20% higher than measured values. Luketic et al. (1993) also found that the StairMaster 4000PT reported METs were higher across multiple stages of an exercise test when compared to measured METs. Because of the differences in equipment used and the conflicting results from previous studies, it is difficult to draw concrete conclusions or make comparisons between the present results and previous research. In general, however, it seems that the stair stepping devices that have been studied do not provide accurate estimates of submaximal oxygen uptake, which reduces their ability to provide accurate predictions of maximal oxygen uptake.

Previous studies have developed equations to predict VO_2max_ based on maximal testing performed on a StairMaster 4000PT (Butts et al., 1993; Holland et al., 1990). That approach might be warranted with stepmills in general and the Matrix C5x specifically. Unfortunately, the present study only completed submaximal exercise on the stepmill, and we were not able to obtain any clarification about the VO_2max_ prediction equation provided by Matrix. Attempts to calculate VO_2max_ using the provided equation and Wattage data from the C5x submaximal tests resulted in VO_2max_ values that were not only not the same as those displayed on the screen, but also not reasonable VO_2max_ values. It is possible that the results from the present study may have turned out differently if we had been able to use the Matrix prediction equation to calculate VO_2max_ values by hand. In an effort to find another way that VO_2max_ might be calculated using submaximal data from the stepmill, submaximal VO_2_ was calculated using the Holland et al.’s (1990) equation, which uses stepmill height and a stepmill stepping rate to determine oxygen uptake (and therefore METs) for a given stage. The ACSM stepping equation includes a stepping rate pattern designed for bench step exercise (i.e. “up up down down”) and so was determined not to be an appropriate way to calculate VO_2_ values from a stepmill where stepping only occurs in one direction (Holland et al., 1988; Luketic et al., 1993). The calculated submaximal VO_2_ and MET values from the Holland et al.’s (1990) equation showed promise, as the values calculated did not differ significantly from measured MET values in two of the three stages examined. However, when those values were used to calculate VO_2max_ using the multi-stage prediction equation (Gibson et al., 2018; Roy et al., 2004), the mean CalcVO_2max_ value was significantly lower than the measured VO_2max_ value. Nevertheless, the correlation between the two was strong and significant. A review of the literature yielded no other options for predicting VO_2max_ values from submaximal stepmill exercise, although a recent study found that during treadmill exercise, the accuracy of the multi-stage equation to estimate VO_2max_ was improved when submaximal VO_2_ used in the equation was measured rather than predicted (Peterman et al., 2021). Using measured submaximal VO_2_ might improve the accuracy of stepmill-based VO_2max_ predictions as well, particularly as the ACSM stepping MET equation may not be applicable to stepmill exercise, and the Holland et al.’s (1990) equation has not been validated. However, measuring VO_2_ may not be feasible in health-fitness settings, so there is a need to develop an equation accessible to the general population that can accurately calculate VO_2max_ values from stepmill exercise.

This study was limited by a small and convenient sample. Due to this, comparisons were not made between males versus females or trained versus untrained participants. The Matrix C5x also did not function as intended, with the preprogrammed submaximal test failing a majority of the time. The Holland et al.’s (1990) equation that we used in our calculations has not been validated, and thus may be considered a limitation. A major strength of this study is that it is a unique assessment of stepmill exercise and provides information on which to build future studies.

In summary, the present study’s findings align with previous research in that stair stepping devices do not provide accurate submaximal MET estimation. Most of the time, METs were overestimated. This can be misleading when estimating intensity or submaximal and maximal VO_2_. If METs are overestimated, intensity and, thus, energy expenditure will be overestimated as well. If this equipment is used in fitness testing, the tester should be aware that the display may not reflect the user’s actual level of exertion. An inability to accurately estimate submaximal intensity also impacts the device’s ability to accurately estimate VO_2max_. The present study did also find that the Matrix C5x stepmill did not provide an accurate estimate of maximal oxygen uptake and that the equation provided by the manufacturer was not useful for calculations by hand. With the growing popularity of stepmills like the C5x, and given this specific device provides a programmed submaximal test, the equations and methods behind submaximal testing on these devices need to be further researched.
